# Evaluation of treatment patterns, healthcare resource utilization and cost of illness for sickle cell disease in Ghana: a private medical insurance claims database study

**DOI:** 10.1186/s12913-023-09984-6

**Published:** 2023-09-21

**Authors:** Kwaku Marfo, Yvonne Dei-Adomakoh, Catherine Segbefia, Duah Dwomoh, Adeline Edgal, Nancy Ampah, Badarinath Chickballapur Ramachandrachar, Kumaresan Subramanyam, Ashok Natarajan, Olufolake Egbujo, Kenneth I. Ataga

**Affiliations:** 1 Novartis Pharma AG, Basel, Switzerland; 2 University of Ghana Medical School, Korle Bu Teaching Hospital, Accra, Ghana; 3 University of Ghana Legon, Accra, Ghana; 4 Novartis Ghana Limited, Accra, Ghana; 5 Nationwide Medical Insurance, Accra, Ghana; 6 IQVIA, 11th Floor Convention Tower, DWTC, Al Saada Street, Dubai, 33083 UAE; 7 IQVIA, Bengaluru, India; 8 Novartis Pharmaceuticals Corporation, East Hanover, USA; 9 University of Tennessee Health Science Center, Memphis, USA

**Keywords:** Sickle cell disease, Vaso-occlusive crisis, Healthcare resource utilization, Hydroxyurea, Claims database

## Abstract

**Background:**

Sickle cell disease (SCD) is a major public health concern in sub-Saharan Africa, accounting for nearly 75% of the global disease burden. The current analysis evaluated patient characteristics, treatment patterns, healthcare resource utilization (HCRU) and associated costs in patients with SCD based on a Private Medical Insurance Database in Ghana.

**Methods:**

This retrospective longitudinal cohort study was conducted using an e-claims database from Ghana (01 January 2015 to 31 March 2021). Patients were stratified by age (0 month to < 2 years, ≥ 2 years to ˂6 years, ≥ 6 years to < 12 years, ≥ 12 years to < 16 years; ≥16 years), vaso-occlusive crisis (VOC) (< 1, ≥ 1 to < 3, and ≥ 3 per year), and continuous enrolment. Study outcomes related to patient characteristics, comorbidities, treatment pattern, HCRU were evaluated for pre- and post-index period (index period was between July 2015 to March 2020). Descriptive analysis was used to analyse different study variables.

**Results:**

The study included 2,863 patients (mean age: 20.1 years; Min age: 0; Max age: 83; females 56.1%). Overall, 52.2% (*n* = 1,495) of SCD patients were ≥ 16 years and 17.0% (*n* = 486) were in the ≥ 2 to ˂6-years age group. The majority of patients aged ≥ 16 years (62.5%) in the database did not have reported VOC episodes, 35.9% of patients had 1 to 3 VOCs per year and 1.5% had ≥ 3 VOCs per year during the follow-up period. Consultation-based prevalence of SCD was 0.5% [95% confidence interval (CI): 0-1.3%] − 1.4% [CI: 0.6-2.2%]. Malaria, upper respiratory tract infection (URTI) and sepsis were the common complications of SCD. Analgesics were the most frequently prescribed medications followed by anti-infectives, hematinics, and antimalarials. Hydroxyurea, a routine standard of care for SCD was under-utilized. SCD patients had median cost incurred for consultation/hospital services of $11.3 (Interquartile range [IQR] $6.2 - $27.2). For patients with VOC, maximum median cost was incurred for medications ($10.9 [IQR $5.0-$32.6]). Overall median healthcare cost was highest for individuals with ≥ 3 VOCs per year during the follow-up period ($166.8 [IQR $70.3-$223.5]).

**Conclusion:**

In this retrospective private insurance claims database analysis, SCD imposes a significant healthcare burden, especially in patients with VOC. There is a need for reimbursed treatment options that could reduce the long-term burden associated with SCD and VOC.

**Supplementary Information:**

The online version contains supplementary material available at 10.1186/s12913-023-09984-6.

## Background

 Sickle cell disease (SCD) is a group of monogenetic autosomal recessive disorders attributable to point mutations in the beta-globin gene [1, 2]. SCD leads to progressive organ dysfunction and failure due to vaso-occlusion, a chronic inflammatory state and endothelial dysfunction [1, 3]. There are many SCD genotypes, with the most common in sub-Saharan Africa being homozygous SCD and compound heterozygous β-globin S and β-globin C [4]. SCD is the most frequent genetically inherited life-threatening disorder, primarily affecting the African, Indian, Caribbean, and Arab populations [5]. According to the systematic analysis of the Global Burden of Disease Study, 3.2 million people are affected by SCD globally and 176,000 people die per year due to SCD-associated complications [3]. The highest prevalence of the disease is in sub-Saharan Africa where 240,000 people are born with SCD annually [6] and mortality rate is high, with an estimated 50–90% of children dying before the age of 5 [4]. In Ghana, SCD accounts for about 6.3% of the total disease burden in sub-Saharan Africa, with nearly 2% of neonates (~ 15,000) being diagnosed with SCD annually [7].

The clinical manifestations of SCD are broad, and include vaso-occlusive crisis (VOC), hemolytic anemia, acute aplastic crisis, infections including bacterial sepsis, respiratory complications, acute splenic sequestration, and psychosocial morbidity [5, 8]. The burden of malaria is high in sub-Saharan Africa [9]. VOCs are the primary cause of health care encounters in patients with SCD and increased VOC burden is associated with increased risk of mortality [10].

In the management of SCD, hydroxyurea, approved by the United States Food and Drug Administration (USFDA) and European Medicine Agency (EMA), remains a key therapeutic option. In 2018, the Ghana Food and Drug Authority approved the use of hydroxyurea in treatment of both adults and children with SCD [11]. Hydroxyurea exhibits multimodal action – it induces fetal hemoglobin production and reduces inflammation and cell adhesion. Long-term use of hydroxyurea is safe and effective in both pediatric and adult SCD patients [12]. However, laboratory monitoring (complete blood count with white blood cell differential and reticulocyte count) is warranted with the use of hydroxyurea to balance the efficacy and safety risk profile, as hydroxyurea therapy is associated with risk of developing infection, causing myelosuppression and hepatotoxicity [13–15]. In recent years, the treatment landscape of SCD has slowly expanded with the advent of novel therapeutic approaches. These therapeutic options target different categories of pathobiology – (i) inhibition of sickle hemoglobin polymerization (voxelotor), (ii) targeting sickle red blood cells (RBCs) - and white blood cells (WBCs) endothelial adhesive events (crizanlizumab), and (iii) antioxidant agents (L-glutamine) [12]. Other therapeutic agents used in SCD include antibiotics, antimalarial agents or antimalarial prophylactics and blood transfusions [12, 16]. Besides the diagnostic and therapeutic challenges, another major hurdle in the management of patients with SCD is the lack of established practices for transition from pediatric to adult care which adds to the healthcare resource utilization (HCRU) in terms of emergency room visits, higher inpatient stays and acute care visits [2].

Although significant progress has been made in the treatment of SCD, it still imposes a substantial economic burden in terms of cost, HCRU and out-of-pocket expenditure. VOC episodes further add to the economic burden of SCD as they are the most common cause of hospitalization and emergency department visits [17].

In the United States (US), the annual mean cost for SCD treatment was United States dollars [US$] 20,206, the highest in patients with more than 3 VOC episodes [18]. In addition, apart from the direct economic burden, SCD also levies an indirect economic burden in terms of loss in work productivity that amounts to US$ 3,145,862 in adult SCD patients with VOC episodes for a period of one year [19].

Sickle cell disease management requires an integrated and holistic approach. However, there are many challenges with the provision of SCD care in sub-Saharan Africa, such as lack of newborn screening, and diagnostic tools, inequities in access to healthcare, including novel therapeutic options, absence of standard treatment guidelines, significant stigmatization, the need for well-resourced and equipped laboratories, few dedicated SCD treatment centers, lack of appropriate preventive and curative services, lack of disease awareness, weak public health systems, financial and emotional cost burden [20, 21].

According to World Bank classification, Ghana is categorized as a lower middle-income country, with incidence of poverty being 45.6% [22]. In the year 2016 (the most recent year for which World Bank estimates are currently available), the poverty headcount ratio at $2.1 a day was 25.3% in the Ghanaian population [23]. As per recent reports, the gross living wage per month in Ghana is $257, while the minimum wage has remained $2 past 2 decades [24]. In a study conducted by the Consortium for the Advancement of Sickle Cell Research, SCD patients in Ghana visited emergency or outpatient departments more frequently for their pain episodes (annualized mean 2.0) compared to SCD patients in the US, United Kingdom (UK), and Italy [25]. In a country like Ghana with high poverty levels, frequent hospitalization and other healthcare expenditures associated with SCD, can impose a substantial economic burden on the affected families and healthcare facilities. In this context, the current study was conducted to determine the treatment pattern and financial burden of SCD in Ghana. The aim of the current analysis was to evaluate the patient characteristics, treatment patterns, hospitalizations, HCRU, total cost incurred in patients with SCD and its subsets, based on the analysis of a private medical insurance (Nationwide Medical Insurance) database in Ghana.

## Methods

### Study design

This was a longitudinal, secondary database, retrospective, single-cohort study performed using insurance claims data from January 1, 2015 to March 31, 2021. The index date for each patient was defined as the date of first record of diagnosis of SCD, identified during the index period (from 01 July 2015 to 31 March 2020) in the database. Patients with at least 1 SCD-related diagnosis claim in the index period and a minimum follow-up period of 12 month were included in the study. The pre-index period (refers to specified period prior to index date i.e., 6-month baseline before the index period) and post-index period (refers to specified period after index date i.e., 12-month follow-up after the index period) were required to assess the study outcomes.

### Data source

The Nationwide Medical Insurance Claims Database is an anonymized patient-level database of all insurance claims generated from their subscribers across Ghana. The database includes manually processed claims from the hospital. Each patient has a unique identifier (ID) in the database and using this ID, patients are linked to multiple visits and other services.

In the current study’s context, the database provided comprehensive data on patient demographics (age and sex), date of attendance, encounter type (inpatient or outpatient), activity type (drugs, consumables [medical/surgical supplies], procedures [medical/surgical procedures or diagnostic investigations], services [consultation or hospital services] and others [administrative services]), claimed amount, reimbursed amount, and diagnostic information.

### Study population

The study population was identified based on International Classification of Diseases, Tenth Revision, Clinical Modification (ICD-10-CM) codes (ICD-10 codes for diagnosis of SCD [D57: Sickle cell disorders; D57.0: Sickle cell crisis/VOC with acute chest syndrome; D57.1: Sickle cell anemia without crisis; D57.2: Double heterozygous sickling disorders; and D57.8: Other sickle cell disorders]). The study population was stratified by age group (0 month to < 2 years, ≥ 2 years to ˂6 years, ≥ 6 years to < 12 years, ≥ 12 years to < 16 years; ≥16 years). Sub-group analysis was also conducted based on the number of episodes of VOC per year and VOC claims among SCD patients aged ≥ 16 years (< 1 VOC episode per year, ≥ 1 to < 3 VOC episodes per year, ≥ 3 VOC episodes per year). In the study, discrete VOC-related claims within a 3-day gap were combined and recorded as a single VOC episode. VOC episodes were identified based on ICD-10 codes (D57.0, D57.1, D57.2) or prescription of medications (non-steroidal anti-inflammatory drugs [NSAIDs]/Opioids).

The study included patients with follow-up data of at least 12 months, for ≥ 1 diagnosis claims with SCD during the index period. Continuous medical enrolment data for at least 1 claim (any service) during the 6-month baseline period and 12-month follow-up period were assessed. There were no explicit exclusion criteria for patient selection in the study; however, the patients with no follow-up data or continuous medical enrolment data were excluded. Out of 3,980 potentially eligible patients identified from the database, 2,863 (71.9%) patients met the inclusion criteria and were included in the analysis.

### Ethical considerations

Ethics committee approval or obtaining informed consent from patients was not required for this study as it did not involve the collection, use or transmittal of individual identifiable data. Patient identity or medical records were not disclosed in this study. The database uses anonymized data. Anonymization refers to the process of protecting private or sensitive information of patient by complete removal of identifiers such as name of the patient, date of birth, email address etc., that can connect the individual to the data. The study conformed to the ethical principles outlined in the Good Pharmacoepidemiology Practices Guidelines, Declaration of Helsinki 1964 and its later amendments, Good Epidemiological Practice guidelines issued by the International Epidemiological Association, Good Practices for Outcomes Research issued by the International Society for Pharmacoeconomics and Outcomes Research and other applicable guidelines/laws. The dataset and the security of the office where the data set were kept met the requirements of the Health Insurance Portability and Accountability Act of 1996.

The data used for the current study was not publicly available. This data was obtained from Nationwide Medical Insurance following all necessary legal procedures to acquire and use this anonymized patient claims data.

### Study outcome measures

- Demographics and patient characteristics comprising of age, gender and VOC episodes per year were extracted from the claim records.
- The consultation-based prevalence was calculated for the patients with SCD and those with VOC during the study period (01 January 2015 to 31 March 2021) considering the overall population registered in Nationwide’s Database.
- Number of patients with each SCD genotype were analyzed based on the ICD-10-CM codes, by year and overall time periods.
- The comorbidities/complications were assessed during the study period for patients based on age groups and having ≥ 1 SCD claim in the index period.
- Prescription patterns of SCD treatment (including VOC crisis) in terms of number of patients and claims were evaluated based on different drug categories.
- The time to first hospitalization (refers to the period from index date to first hospitalization date) during the study period and annual rate of hospitalization during the 12-month follow-up period were also assessed. The assessment was done for both SCD and VOC episodes based on the ICD-10-CM codes for VOC episodes (D57.0, D57.1 and D57.2).
- Claims data for patients were analyzed for baseline all-cause, SCD-related and VOC–related HCRU, and associated costs (all-cause costs refers to the healthcare costs incurred for all the claims that the patient encountered during the follow-up period; SCD-related and VOC-related costs refers to the healthcare costs incurred for claims that were specific to SCD and VOC, respectively, during the follow-up period) in a 12-month follow-up period from the index diagnosis date (IDD), based on visit type (inpatient and outpatient) and activity type (drugs, consumables, procedures, services, and others). The patients were not mutually exclusive. All-cause HCRU and associated costs were also analyzed for the 6-month baseline period prior to the IDD, only by visit type. The overall HCRU and associated costs were calculated for the VOC-related claims by the adolescent and adult SCD patients (≥ 16 years). This was for the 12-month follow-up period from the IDD and by the VOC episodes.
- Number and percentage of SCD patients visiting different specialties were evaluated based on claims during the study period after the IDD.
- Different diagnostic methods, procedures and techniques used in the management of SCD during the study period were assessed based on the number and percentage of patients who underwent the diagnostic tests.

### Statistical analysis

Descriptive analysis was used to analyze the different study variables throughout the pre-index and post-index periods. Continuous variables were summarized as number of observations, mean, standard deviation, median, interquartile range (IQR), minimum and maximum, as appropriate. Categorical variables were summarized as frequency and percentages (n, %), and by subgroups of VOC episodes, where appropriate. Demographics, prevalence estimates and SCD types were reported with 95% confidence interval (CI).

## Results

### Demographics and baseline characteristics of patients with SCD

The mean age of eligible patients was 20.1 ± 16.0 years with the majority being females (*n* = 1,607, 56.1%). About 52.2% (*n* = 1,495) of the patients belonged to the age group of ≥ 16 years (Table 1). With respect to VOC episodes in adolescent and adult patients (*n* = 1,495), 935 patients (62.5%) had zero VOC episodes per year, 537 patients (35.9%) had ≥ 1 to < 3 VOC episodes per year, while 23 patients (1.5%) had ≥ 3 VOCs episodes per year. At least 1 episode of VOC was reported by a total of 1,211 SCD patients and around half (46.2%) of these patients were aged ≥ 16 Years (Table 2).

**Table 1:** Demographic characteristics of patients with SCD (January 2015-March 2021)

Baseline Characteristics	Patients With SCD	
	N	% of Patients [95% CI]
Overall Study Population	2863	
**N (Patient Counts)**	2863	
**Mean (SD)**	20.1 (16.0)	
**Median (IQR)**	22.3 (3.9, 32.7)	
**Age (As at Index Date) - Number of Patients**		
0 months to < 2 years	459	16.0% [13-19%]
≥ 2 years to ˂6 years	486	17.0% [17-20%]
≥ 6 years to < 12 years	306	10.7% [7-14%]
≥ 12 years to < 16 years	117	4.1% [0-8%]
≥ 16 years	1495	52.2% [50-55%]
**Sex - Number of Patients**		
Male	1256	43.9%
Female	1607	56.1%

*Abbreviations*: *CI *Confidence interval, *IQR *Interquartile range, *N *Number of patients, *SCD *Sickle cell disease, *SD *Standard deviation

**Table 2:** Age distribution of SCD patients with VOC (January 2015-March 2021)

SCD Patients with VOC (Period: 1-year Follow-up from Index Date of SCD Diagnosis)		
Age	N	% of Patients [95% CI]
**≥ 16 years**	**1495**	
< 1 VOC per year (no VOC episodes)	935	62.5% [59-66%]
≥ 1 to < 3 VOCs per year	537	35.9% [32-40%]
≥ 3 VOCs per year	23	1.5% [0-7%]
**VOC patients with Either VOC Diagnosis Codes or VOC Drugs**	1211	
0 months to < 2 years	183	15.1% [10-20%]
≥ 2 years to ˂6 years	260	21.5% [16-26%]
≥ 6 years to < 12 years	153	12.6% [7-18%]
≥ 12 years to < 16 years	55	4.5% [0-10%]
≥ 16 years	560	46.2% [42-50%]

*Abbreviations*: *CI *Confidence interval, *N *Number of patients, *SCD *Sickle cell disease, *VOC *Vaso-occlusive crisis

### Consultation-based prevalence of patients with SCD and VOC

**Fig. 1:**
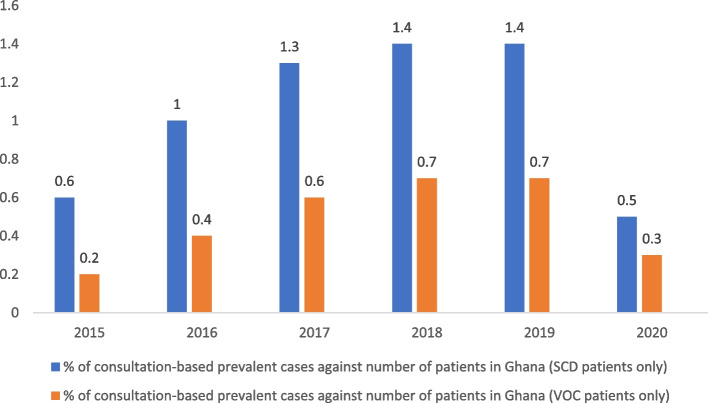
Comparison of consultation-based prevalence in patients with SCD and VOC. Abbreviations: SCD: Sickle cell disease; VOC: Vaso-occlusive crisis

During the study period (January 2015-March 2021), year-on-year prevalence of patients with SCD and VOC were analyzed using the claims database. A steady increase in prevalence of patients with SCD and patients with SCD and VOC episodes was observed from year 2015 (0.6%; [95% CI: 0-1.6%] and 0.2%; [95% CI: 0-1.1%], respectively) till the year 2019 (1.4% [95% CI: 0.6-2.2%] and 0.7% [95% CI: 0-1.5%], respectively). However, a dip in the prevalence to 0.5% [95% CI: 0-1.3%] for SCD and 0.3% [95% CI: 0-1.1%] for SCD patients with VOC episodes was noted for the year 2020 (Fig. 1).

### Types of SCD

The study patients (*n* = 2,863) were categorized based on the clinical type of SCD reported, using the ICD-10-CM diagnosis codes. During the study period (January 2015-March 2021), of the 2,863 study patients, 89% (*n* = 2,562; [95% CI: 88−91%]) of patients reported with diagnosis code for sickle cell disorders (D57), 9% (*n* = 251; [95% CI: 5−12%]) for other sickle cell disorders (D 57.8) and 7% (*n* = 211; [95% CI: 4−11%]) of patients for sickle cell crisis/VOC with acute chest syndrome (D57.0) (Table S1)). It should be noted that patient counts were not mutually exclusive.

### Comorbidities/Complications in patients with SCD

The most common complications in ≥ 16 years age group included malaria (*n* = 1,440; 96.3%), upper respiratory tract infection (URTI) (*n* = 1,071; 71.6%), sepsis/septicemia (*n* = 445; 29.8%), acute chest syndrome (*n* = 214; 14.3%) and cellulitis (*n* = 108; 7.2%), and this trend was similar in patients of ≤ 16 years age group (Table 3). Other complications more commonly observed in the ≥ 16 years age group and not in the younger age groups were end-stage kidney disease requiring dialysis (n = 847; 56.7%) and liver disease (*n* = 336; 22.5%). The most common complications in patients with VOC episodes were malaria (< 1 VOC per year: *n* = 894, 95.6%; ≥ 1 to < 3 VOCs per year: *n* = 523, 97.4%; ≥ 3 VOCs per year: *n* = 23, 100%), URTI (< 1 VOC per year: *n* = 671, 71.8%; ≥ 1 to < 3 VOCs per year: *n* = 383, 71.3%; ≥ 3 VOCs per year: *n* = 17, 73.9%) and end-stage kidney disease requiring dialysis (< 1 VOC per year: *n* = 552, 59.0%; ≥ 1 to < 3 VOCs per year: *n* = 287, 53.4%; ≥ 3 VOCs per year: *n* = 8, 34.8%). Other complications observed in patients having ≥ 3 VOCs per year were gallstones (*n* = 4, 17.4%) and osteomyelitis (*n* = 4, 17.4%).

**Table 3:** Complications/Comorbidities in patients with sickle cell disease (January 2015-March 2021)

Complications/ Comorbidities								
Number of Patients by Comorbidities/ Complications, Age Subgroups and VOC episodes	Age subgroups N (%)					VOC episodes N (%)		
	0 Months to < 2 Years	≥ 2 Years to ˂6 Years	≥ 6 Years to < 12 Years	≥ 12 Years to < 16 Years	≥ 16 Years	< 1 VOC Per Year	≥ 1 to < 3 VOCs per Year	≥ 3 VOCs per Year
**Overall population**	459 (16.0)	486 (17.0)	306 (11.0)	117 (4.0)	1495 (52.0)	935 (62.5)	537 (35.9)	23 (1.5)
**Malaria**	440 (95.9)	483 (99.4)	298 (97.4)	115 (98.3)	1440 (96.3)	894 (95.6)	523 (97.4)	23 (100.0)
**URTI**	412 (89.8)	449 (92.4)	265 (86.6)	85 (72.6)	1071 (71.6)	671 (71.8)	383 (71.3)	17 (73.9)
**Dialysis**	17 (3.7)	24 (4.9)	30 (9.8)	21 (17.9)	847 (56.7)	552 (59.0)	287 (53.4)	8 (34.8)
**Sepsis/Septicemia**	264 (57.5)	277 (57.0)	141 (46.1)	37 (31.6)	445 (29.8)	258 (27.6)	174 (32.4)	13 (56.5)
**Liver**	6 (1.3)	10 (2.1)	7 (2.3)	7 (6.0)	336 (22.5)	210 (22.5)	121 (22.5)	5 (21.7)
**Acute chest syndrome**	140 (30.5)	148 (30.5)	69 (22.5)	18 (15.4)	214 (14.3)	123 (13.2)	83 (15.5)	8 (34.8)
**Cellulitis**	26 (5.7)	34 (7.0)	16 (5.2)	12 (10.3)	108 (7.2)	56 (6.0)	48 (8.9)	4 (17.4)
**Acute kidney disease**	2 (0.4)	3 (0.6)	2 (0.7)	3 (2.6)	58 (3.9)	34 (3.6)	16 (3.0)	3 (13.0)
**Acute kidney injury**	1 (0.2)	6 (1.2)	2 (0.7)	4 (3.4)	53 (3.5)	34 (3.6)	23 (4.3)	1 (4.3)
**Central nervous system**	11 (2.4)	12 (2.5)	6 (2.0)	2 (1.7)	54 (3.6)	29 (3.1)	23 (4.3)	2 (8.7)
**Gall stones**	0 (0.0)	0 (0.0)	3 (1.0)	2 (1.7)	48 (3.2)	20 (2.1)	24 (4.5)	4 (17.4)
**Osteomyelitis**	8 (1.7)	10 (2.1)	7 (2.3)	0 (0.0)	12 (0.8)	7 (0.7)	1 (0.2)	4 (17.4)
**Stroke**	1 (0.2)	0 (0.0)	1 (0.3)	0 (0.0)	9 (0.6)	7 (0.7)	2 (0.4)	0 (0.0)
**Heart failure**	3 (0.7)	3 (0.6)	0 (0.0)	0 (0.0)	18 (1.2)	6 (0.6)	11 (2.0)	1 (4.3)
**Cardiomyopathy**	0 (0.0)	0 (0.0)	0 (0.0)	0 (0.0)	10 (0.7)	4 (0.4)	5 (0.9)	1 (4.3)
**Leg ulcer**	1 (0.2)	3 (0.6)	3 (1.0)	1 (0.9)	12 (0.8)	4 (0.4)	7 (1.3)	1 (4.3)

*Abbreviations*: *N *Number of patients, *SCD *Sickle cell disease, *URTI *Upper respiratory tract infection, *VOC *Vaso-occlusive crisis

### Treatment patterns

Of the total study patients (*n* = 2,863), 1,500 patients (52.4%) were treated with different drug classes. The most common drug classes prescribed to patients were NSAIDs (*n* = 1,134, 75.6%), anti-infectives (*n* = 783, 52.2%), hematinics (*n* = 419, 27.9%), antimalarial (*n* = 398, 26.5%), opioids (*n* = 146, 9.7%), non-opioids (*n* = 19, 1.3%) and hydroxyurea (*n* = 4, 0.3%). Out of 1,497 patients ≥ 16 years of age, 738 patients (49.4%) were treated with different drug classes. The most common drug classes prescribed for patients more than ≥ 16 years were NSAIDs (*n* = 501, 67.9%), anti-infectives (*n* = 308, 41.7%), hematinics (*n* = 271, 36.7%), antimalarial (*n* = 158, 21.4%), opioids (*n* = 117, 15.9%) and non-opioids (*n* = 16, 2.2%) (Table 4).

**Table 4:** Drug/Treatment patterns for patients with SCD (January 2015-March 2021)

	Patients with SCD (Overall population)		Patients with SCD (age ≥ 16 adult population)	
Drug Classes	Patients; N (%)	Claims; N (%)	Patients; N (%)	Claims; N (%)
**Analgesics (NSAIDs)**	1134 (75.6)	1764 (70.4)	501 (67.9)	750 (65.8)
**Anti-infectives**	783 (52.2)	1186 (47.3)	308 (41.7)	428 (37.5)
**Hematinic**	419 (27.9)	837 (33.4)	271 (36.7)	428 (37.5)
**Antimalarial**	398 (26.5)	536 (21.4)	158 (21.4)	217 (19.0)
**Opioids**	146 (9.7)	271 (10.8)	117 (15.9)	208 (18.2)
**Anti-inflammatory**	54 (3.6)	67 (2.7)	20 (2.7)	32 (2.8)
**Antihypertensive/Vasodilators**	46 (3.1)	57 (2.3)	45 (6.1)	56 (4.9)
**Antiasthmatics**	21 (1.4)	21 (0.8)	6 (0.8)	6 (0.5)
**Vaccine**	21 (1.4)	21 (0.8)	20 (2.7)	20 (1.8)
**Non-opioid Analgesics**	19 (1.3)	19 (0.8)	16 (2.2)	16 (1.4)
**Anti-platelet**	17 (1.1)	22 (0.9)	16 (2.2)	21 (1.8)
**Antidepressants**	6 (0.4)	7 (0.3)	6 (0.8)	7 (0.6)
**Anticoagulants**	4 (0.3)	4 (0.2)	3 (0.4)	3 (0.3)
**Hydroxyurea**	4 (0.3)	20 (0.8)	-	-

*Abbreviations*: *N *Number, *NSAID *Non-steroidal anti-inflammatory drugs, *SCD * Sickle cell disease

Numbers are not mutually exclusive

### Hospitalization during 12-Month follow-up period

Overall, 80 patients were hospitalized with 109.5 (IQR 39.0-198.0) median days of time to first hospitalization and a median annual rate of hospitalization of 1.0 (IQR 1.0–2.0). For the patients who experienced VOC episodes (*n* = 37), the median time to first hospitalization was 162.0 (IQR 48.0253.0) days and median annual rate of hospitalization was 1.0 (IQR 1.0–2.0) (Table S2).

### Healthcare resource utilization and associated costs

#### Healthcare resource utilization and cost during the 6-month baseline period by visit type

During the baseline period of 6 months, for patients with SCD, the total number of claims was higher for outpatient visits (3.0 [IQR 2.0–6.0]) compared to inpatient visits (1.0 [IQR 1.0–1.0]), however, the median inpatient cost was higher compared to outpatient cost ($576.4 [IQR $291.9-$1,129.2] and $49.9 ([IQR $25.5-$91.4], respectively). Similarly, for SCD patients experiencing VOC episodes, the median number of claims for outpatient visit was higher than inpatient visits (3.0 [IQR2.0-5.0]) and 1.0 [IQR 1.0–1.0], respectively), while the inpatient cost was higher than outpatient cost ($70.4 ([IQR $36.8-$141.6] and $43.4 [IQR $23.2-$81.2], respectively). Higher overall healthcare costs were incurred by SCD patients compared to SCD patients experiencing VOC episodes ($55.4 (IQR $27.7-$107.9) and $48.8 (IQR $25.0-$100.9), respectively) (Table S3). This could be attributed to the fact that SCD patients group included the overall population while SCD patients experiencing VOC episodes included only the patients above 16 years of age. Additionally, the HCRU cost assessed during the baseline period also included the costs incurred for all comorbidities-related claims for patients with SCD and SCD with VOC; while the HCRU cost evaluated during the follow-up period included only costs specific to SCD and SCD with VOC claims.

#### Healthcare resource utilization and cost during 12-month follow-up period by visit type

Assessment of disease-specific HCRU and associated costs for 12-month follow-up period among all SCD patients showed that despite median HCRU outpatient claims and inpatient claims (1.0 claims) being same, the median healthcare cost incurred due to inpatient claims ($92.6) was higher than the cost of outpatient claims ($25.8). A similar observation was noted in SCD patients with VOC episodes. The median HCRU cost was higher due to the inpatient visits ($95.2) than the outpatient visits ($24.6). A higher median overall cost was noted to be incurred by SCD patients with VOC episode ($32.9) than all SCD patients ($30.8) despite the number of all SCD patients (*n* = 406) being twice as more than the SCD patients with VOC (*n* = 206) (Table 5).

**Table 5:** Healthcare resource utilization and costs by visit type (12-Month Follow-up period)

Sickle Cell Disease		
	**SCD-Related Healthcare Resource Utilization and Cost**	**VOC-Related Healthcare Resource Utilization and Cost**
Number of patients in 12-month follow-up	406	206
Number of claims in 12-month follow-up	971	359
**Healthcare Utilization: Number of Visits (Claims)**		
Overall		
N (patient count)	406	206
Total	971	359
Mean	2.4	1.7
SD	2.8	1.5
Median	1.0	1.0
Minimum	1.0	1.0
Maximum	23.0	11.0
Q1 (lower quartile)	1.0	1.0
Q3 (upper quartile)	2.0	2.0
***Inpatient Visits***		
N (patient count)	80	71
Total	133	120
Mean	1.7	1.7
SD	1.4	1.4
Median	1.0	1.0
Minimum	1.0	1.0
Maximum	8.0	8.0
Q1 (lower quartile)	1.0	1.0
Q3 (upper quartile)	2.0	2.0
***Outpatient Visits***		
N (patient count)	384	71
Total	838	120
Mean	2.2	1.7
SD	2.4	1.4
Median	1.0	1.0
Minimum	1.0	1.0
Maximum	21.0	8.0
Q1 (lower quartile)	1.0	1.0
Q3 (upper quartile)	2.0	2.0
**Healthcare Costs (Reported in US$)**		
*** Overall costs***		
N (patient count)	406	206
Total	31891.1	19995.7
Mean	78.6	97.1
SD	185.0	233.0
Median	30.8	32.9
Minimum	0.0	0.0
Maximum	2029.8	2000.1
Q1 (lower quartile)	17.6	20.1
Q3 (upper quartile)	74.5	91.0
*** Inpatient Cost***		
N (patient count)	80	71
Total	15635.4	14400.5
Mean	195.4	202.8
SD	344.3	360.2
Median	92.6	95.2
Minimum	14.7	18.1
Maximum	2000.1	2000.1
Q1 (lower quartile)	49.4	51.8
Q3 (upper quartile)	188.2	177.6
*** Outpatient Cost***		
N (patient count)	384	174
Total	16255.7	5595.2
Mean	42.3	32.2
SD	45.1	26.5
Median	25.8	24.6
Minimum	0.0	0.0
Maximum	293.6	169.3
Q1 (lower quartile)	15.3	16.7
Q3 (upper quartile)	52.0	35.5

Abbreviations: N: Number of patients; SCD: Sickle cell disease; SD: Standard deviation; US$: United States dollar; VOC: Vaso-occlusive crisis

Note: Currency conversion rates

Source for conversion of Cedi to US$ currency: https://www.unitconverters.net/currency/ghs-to-usd.htm; Accessed on 11August 2022 14:52:0

1 Ghanaian Cedi=0.1132 United States dollar (currency values in US$ rounded off to one decimal point)

Q1 (lower quartile) - Q1 is the median (the middle) of the lower half of the data

Q3 (upper quartile) - Q3 is the median (the middle) of the upper half of the data.

#### Healthcare resource utilization and cost during 12-month follow-up period by activity type

The median number of claims was same for different activity types for SCD patients and SCD patients with VOC episodes. In patients with SCD, the highest cost incurred was for consultation or hospital services ($11.3 [IQR $6.2-$27.2]) and lowest cost was for administrative services ($5.1 [IQR $2.4-$13.8]). However, for patients with VOC episodes, cost incurred was highest for medications ($10.9 [IQR $5.0-$32.6]) and lowest for administrative services ($6.8 [IQR $2.8-$13.8]) (Table S4). Availability of free health care in the USA or Europe compared to sub-Saharan Africa could explain overall reported relative higher rates of hospital admission versus VOC managed in an outpatient setting [26].

#### By VOC episodes and age groups: for 12-month follow-up

In terms of VOC episodes, maximum number of visits was reported for patients with ≥ 1 to < 3 VOC episodes per year (*n* = 216); however, median was higher in case of ≥ 3 VOCs per year (4.0 [IQR 2.0–7.0]). The median healthcare cost was highest for patients with ≥ 3 VOCs per year ($166.8 [IQR $70.3-$223.5]) (Table 6).

The healthcare costs pertaining to visit type and activity type in SCD patients were higher compared to those costs in SCD patients with VOC episodes, during the 12-month follow-up period.

**Table 6:** Healthcare Resource Utilization and Costs by VOC Episodes and Age Groups (12-Month Follow-up)

VOC-Related Healthcare Resource Utilization and Cost			
	< 1 VOC per Year	≥ 1 to < 3 VOCs per Year	≥ 3 VOCs per Year
	≥ 16	≥ 16	≥ 16
**Healthcare Utilization: Number of Visits (Claims)**			
Overall			
N (patient counts)	61	110	23
Total	101	216	127
Mean	1.7	2.0	5.5
SD	1.5	1.8	4.7
Median	1.0	1.0	4.0
Minimum	1.0	1.0	2.0
Maximum	8.0	10.0	23.0
Q1 (lower quartile)	1.0	1.0	2.0
Q3 (upper quartile)	1.0	2.0	7.0
**Overall Healthcare Costs (Reported in US$)**			
N (patient counts)	61	110	23
Total	3077.8	8374.1	4891.2
Mean	50.5	76.1	212.7
SD	82.1	198.9	305.1
Median	21.8	34.1	166.8
Minimum	0.3	0.0	18.3
Maximum	554.5	2029.8	1535.8
Q1 (lower quartile)	11.8	20.9	70.3
Q3 (upper quartile)	49.6	74.5	223.5

*Abbreviations*: *N *Number of patients, *SD *Standard deviation, *VOC *Vaso-occlusive crisis, *US$ *United States dollar

Currency conversion rates

Source for conversion of Cedi to US$ currency: https://www.unitconverters.net/currency/ghs-to-usd.htm; Accessed on 11August2022 14:52:0

1 Ghanaian Cedi=0.11328 United States dollar (currency values in US$ rounded off to one decimal point)

Q1 (lower quartile) - Q1 is the median (the middle) of the lower half of the data

Q3 (upper quartile) - Q3 is the median (the middle) of the upper half of the data

### Specialties visited by patients with SCD

Of the total 2,863 patients in the study, 2,408 patients visited physicians of different specialties for treatment advice. General physician (GP) visits were the highest with patient visits of 1,810 (75.2%) and claims of 2,867 (69.7%). This was followed by pediatric and gynecology consultations (Table S5). Around 5.7% (*n* = 137) patients had specialist consultation with a total of 252 claims (6.1%).

### Diagnostic investigations

Of the overall study population (*n* = 2,863), 96% of patients (*n* = 2,759) underwent diagnostic investigations with total 4,185 claims. The most frequently used diagnostic investigations with the highest number of claims were full blood count, hemoglobin electrophoresis, blood smear for malaria parasites, sickling test, and routine urine examination (Table S6).

## Discussion

SCD is a global public health concern due to substantial morbidity, mortality, and socioeconomic burden. The sub-Saharan Africa region accounts for more than 75% of global SCD burden [20, 21, 27–29]. Despite this considerable disease burden, there is a dearth of real-world data published on the disease burden, treatment patterns and economic burden of SCD from the sub-Saharan Africa region and could be attributed to the lack of a national database on patients with SCD care in regions of sub-Saharan Africa [30]. Hence, this cost of illness study was conducted to evaluate the HCRU for patients with SCD and VOC episodes in Ghana using Nationwide Medical Insurance Database. The study also assessed diagnostic investigations, treatment patterns and specialties visited for treatment advice among patients with SCD and VOC, in a real-world setting.

The yearly prevalence of patients with SCD as well as the SCD patients with VOC episodes during the study period exhibited an increasing trend, suggestive of the growing disease burden in Ghana. However, a dip in prevalence was observed in the year 2020 which could be attributed to the under-reporting of the claims due to coronavirus disease (COVID-19) pandemic. Majority of the patients were ≥ 16 years of age (52.2%) with a predominantly female population and having VOC episodes. These findings are consistent with the real-world study by Foundation for Sickle Cell Disease Research [31]. In the current study, 33% in of the patients belonged to 0 months to < 6 years age group. A systematic review and meta-analysis reports highest prevalence and mortality of SCD in children under 5 years of age, in Africa [32].

In the current study, number of VOC episodes were evaluated only in patients with SCD aged ≥ 16 years and not in patients with SCD < 16 years. The probable reasons could be difficulty in characterizing VOC episodes in children. Diagnosis of VOC in children is complicated by the fact that VOC affecting the bone is the most common acute clinical manifestation of SCD in this population. Furthermore, VOCs are often confused with the much less frequently occurring osteomyelitis [33, 34]. A recent Phase 3 study reported that a shorter duration of VOC episode was observed in patients < 16 years of age [35]. Additionally, the grouping was done in line with the previous trials conducted on patients with SCD (SOLACE trial and SUSTAIN trial [36, 37]. Moreover, the demographic data obtained during the analysis also represented the high disease burden in patients < 16 years of age (47.8%). Hence, the patients were stratified into subpopulation < 16 years and ≥ 16 years.

In the current study, majority of SCD patients had common sequelae of SCD – VOC episodes, infections, malaria, sepsis, and acute chest syndrome among others in accord with other studies [5, 8]. Furthermore, regions of sub-Saharan Africa with high incidence of SCD are also associated with highest density of malaria. Although the sickle cell mutation at one allele of beta-globin gene confers a survival advantage in malaria endemic areas, inheritance of the mutation at both alleles, predisposes individuals to severe malaria and increased mortality from other complications of SCD [38]. Studies have reported that VOC in SCD patients accounts for 95% of emergency department visits and inpatient admissions [34, 39, 40]. This analysis also evaluated the annual rate of hospitalization admissions which was found to be 1.6 ± 1.1. A study conducted in Ghana on patients (with SCD), aged > 13 years reported the annual hospitalization rate to be 2.6%, attributed mostly to the acute complications [27].

Our analysis identified a greater number of prescriptions for analgesics, anti-infectives, and hematinics as compared to hydroxyurea, the standard of care treatment for SCD. Despite hydroxyurea being included in the World Health Organization model list of essential medications for children and the large body of evidence for its established efficacy in terms of reduced frequency of VOC episodes, transfusions, and hospitalizations [41–44], its use is sub-optimal in the sub-Saharan African region. Minimal use of hydroxyurea in the region could be attributed to lack of data on magnitude and impact of SCD in the region, lack of evidence-based guidelines on feasibility, safety, and benefits of hydroxyurea in the region. Other barriers to use of hydroxyurea in the region include drug availability, cost of treatment and laboratory monitoring. Another study in the region emphasized on wider access to hydroxyurea which provides a cost-effective therapeutic approach to deter the progression of disease in SCD patients [45, 46]. Through public-private partnerships, Novartis is working with Ministry of Health of Ghana, Ghana Health Service, Sickle Cell Foundation and Patient Association Groups to increase access to hydroxyurea including a pediatric-friendly formulation.

Comprehensive management of SCD requires effective healthcare systems and necessitates healthcare professionals to have appropriate knowledge of the disease. The current study noted that the treatment of SCD was largely by GP practice compared to specialized care [47]. Considering the limited number of specialist physicians such as hematologists and pediatricians in the region, upskilling community practice physicians such as GPs and internal medicine doctors could help strengthen the care delivery for SCD patients.

In the current study, cost due to inpatient claims for SCD patients was significantly higher compared to cost due to outpatient claims; the cost incurred on consultation/hospital services was highest compared to other HCRU costs (drugs, consumables, procedure, and administrative services). The SCD patients who experienced ≥ 3 VOC episodes per year incurred nearly 3 times more healthcare costs than those with lesser VOC episodes per year [48]. A retrospective cohort analysis of patient-level data from Medicaid database reported that patients with > 3 VOC episodes had the highest annual SCD cost across all settings (mean: US$ 58,950); suggesting that HCRU costs increased with the increase in number of VOC episodes [18]. Few studies which have assessed the economic burden of SCD in countries within sub-Saharan Africa show that households spend a considerable proportion of their income on direct costs (hospitalization, medications, outpatient visits) associated with SCD [49]. The Economic Intelligence Unit 2020 reported that Nigeria had the highest annual costs (direct and indirect costs) of SCD (US$ 6.5 billion per year), followed by Angola (US$ 719.6 million per year) and Ghana (US$ 472.8 million per year) among other sub-Saharan countries [50]. The report highlighted that in Ghana, medication was the major cost driver for patients aged 5–14 and ≥ 15 years (US$ 346 per year and US$ 526 per year, respectively) [51].

Our study noted higher healthcare costs incurred by SCD patients with VOC episodes than SCD patients without VOC episodes, despite the number of SCD patients without VOC being twice as many as SCD patients with VOC.

Our major limitation for this analysis is based on its retrospective design and analysis from a claims database. The study sample size includes only a subset of the privately insured population of Ghana with continuous enrolment during the study period whose demographic data mapping was available; thus, the results might not be applicable for the entire population with SCD in Ghana. Analysis of Ghana Demographic health survey dataset suggested that economic and educational status, marital status (single/widowed), and geographical disparity are the major determinants for not enrolling in the private insurance in Ghana. The financial status determined the insurance status, with poorest, poorer, and middle-income groups being less likely to pay themselves for insurance. Women (single/widowed) were less likely to be covered relative to married women although this group was more likely to pay the insurance premiums themselves. Geographic disparities and educational status also accounted for inequity in the insurance enrolment [52]. Clinical and disease-specific parameters (e.g., duration or severity of the disease) could not be obtained from the claims data. Emergency department visits have not been included or may have been combined with the inpatient visit, leading to information bias. There could be a possibility of missing data due to patients not following up at the same hospital or group of hospitals or not being able to follow-up at all. The medications filled over the counter, provided by the physicians or obtained out-of-pocket could not be ascertained in the claims data. Only direct healthcare costs were evaluated; indirect costs such as work productivity and quality of life were not analyzed for SCD. The database also does not allow one-to-one mapping between diagnosis and medications/procedures/consumables. Hence, some of the reported SCD specific medications, services and costs may not be related directly to SCD. VOC admission within a gap of 3 days was taken as a single episode to validate the accurate capture of VOC episode and to overcome under- or over-representation of VOC episodes. Additionally, VOC episodes managed at home were not captured. Moreover, VOC data for children under 16 years of age were not captured during the analysis. Our study was designed to capture only VOC data above 16 years of age. The high prevalence and mortality rate associated with children has been noted in previous studies [53, 54]. Hence the VOC sub-group in our study cohort could have been under-represented which has implications on the cost distribution and our findings around this major complication of SCD.

However, the strength of the study lies in the fact that it is the first-of-its-kind being conducted in Ghana using the National Private Insurance Claims database. It captures information for the population covered by Nationwide Medical Insurance Claims Database representing 10–15% of the Ghanaian population.

## Conclusion

To the best of our knowledge, the current study is the first-of-its-kind analysis conducted using e-claims database to evaluate the disease and economic burden on patients with SCD in Ghana. The study findings indicate an increased disease burden on patients with SCD and a substantial impact on the healthcare costs, especially in the sub-group of patients with VOC episodes. In our study VOC episodes were seen in less than half of the SCD patients. The most common complications were malaria, URTI and sepsis. Hydroxyurea use was sub-optimal in this cohort of patients. We hypothesized that barriers relating to drug availability, cost of treatment, and laboratory monitoring could have contributed although this is a cohort of patients with private health insurance. Understanding these barriers and efforts to increase access both in private and public sectors of Ghana is paramount for reducing the disease and economic burden associated with SCD. The study also reported a significantly higher HCRU in the treatment of VOC. The VOC cost burden equals the cost burden of SCD treatment, which warrants the need for better management of VOC. However, the limitations of the current study, as listed above, make it likely that the costs of SCD in Ghana are higher than the cost estimates stated here. Additionally, the direct medical expenses and societal costs have disproportionately larger impact on low-income families.

In 2021, the Ghana Food and Drug Authority approved registration of crizanlizumab for prevention of VOC in SCD patients >16 years and over. Additionally, Ghana is participating in the ongoing Phase 3 clinical trial (STAND) with crizanlizumab, the first time a clinical study has been conducted with a biologic agent for managing SCD. Innovative treatments with disease modifying modalities such as crizanlizumab, targeting VOC episodes could be considered in SCD patients who are intolerable to hydroxyurea or with recurring VOCs despite maximum tolerable doses of hydroxyurea.

### Supplementary Information

** Additional file 1: Table S1.** Type of Sickle Cell Disease (January 2015-March 2021). **Table S2.** Hospitalization in All the Patients Having SCD Diagnosis and Inpatient Visit (12-Month Follow-up period). **Table S3.** Healthcare Resource Utilization and Costs by Visit Type (6-Month Baseline Period). **Table S4.** Healthcare Resource Utilization and Costs by Activity Type (12-Month Follow-up). **Table S5.** Specialities Visited by Patients with SCD (January 2015-March 2021). **Table S6.** Diagnostic Investigations Conducted on Patients With SCD (January 2015-March 2021)

## Data Availability

All data generated or analyzed during this study are included in this published article/as supplementary information files.

## Abbreviations

SCD: Sickle cell disease
VOC: Vaso-occlusive crisis
USFDA: United States Food and Drug Administration
EMA: European Medicine Agency
RBC: Red blood cell
WBC: White blood cell
HCRU: Healthcare resource utilization
US: United States
US$: United States dollar
UK: United Kingdom
ID: Identifier
ICD-10-CM: International Classification of Diseases, Tenth Revision, Clinical Modification
NSAID: Non-steroidal anti-inflammatory drugs
IDD: Index diagnosis date
IQR: Interquartile range
CI: Confidence interval
N: Number of patients
URTI: Upper respiratory tract infection
GP: General physician
COVID-19: Coronavirus disease
