# Supplementary material for: Evaluation of treatment patterns, healthcare resource utilization and cost of illness for sickle cell disease in Ghana: a private medical insurance claims database study
Source: BMC Health Serv Res. 2023 Sep 21;23:1018. doi: 10.1186/s12913-023-09984-6 (PMC10515235; doi:10.1186/s12913-023-09984-6)
Supplement: Supplementary file 1 — Additional file 1: Table S1. Type of Sickle Cell Disease (January 2015-March 2021). Table S2. Hospitalization in All the Patients Having SCD Diagnosis and Inpatient Visit (12-Month Follow-up period). Table S3. Healthcare Resource Utilization and Costs by Visit Type (6-Month Baseline Period). Table S4. Healthcare Resource Utilization and Costs by Activity Type (12-Month Follow-up). Table S5. Specialities Visited by Patients with SCD (January 2015-March 2021). Table S6. Diagnostic Investigations Conducted on Patients With SCD (January 2015-March 2021) [file 12913_2023_9984_MOESM1_ESM.docx]

**Supplementary Tables**

**Table S1: Type of Sickle Cell Disease (January 2015-March 2021)**

| **Diagnosis Code** | **Diagnosis Name** | **2015** | **2016** | **2017** | **2018** | **2019** | **2020** | **2021 (Jan 2021-Mar 2021)** | **Overall % (Jan 2015-Mar 2021)** | **Overall Number (Jan 2015-Mar 2021)** | **95% Confidence**  **Interval** |
| --- | --- | --- | --- | --- | --- | --- | --- | --- | --- | --- | --- |
| D57 | Sickle cell disorders | 95.0% | 95.0% | 93.5% | 89.4% | 80.2% | 82.0% | 91.5% | **89%** | 2562 | [88%-91%] |
| D57.0 | Sickle cell crisis/VOC with acute chest syndrome | 7.1% | 7.6% | 7.5% | 7.0% | 9.0% | 12.4% | 20.3% | **7%** | 211 | [4%-11%] |
| D57.1 | Sickle cell anemia without crisis | 1.2% | 0.8% | 3.0% | 3.8% | 5.4% | 9.9% | 10.2% | **4%** | 113 | [0%-8%] |
| D57.2 | Double heterozygous sickling disorders | 0.0% | 0.0% | 0.3% | 0.1% | 0.1% | 0.4% | 0.0% | **0%** | 5 | [0%-4%] |
| D57.8 | Other sickle cell disorders | 0.0% | 1.0% | 2.0% | 7.9% | 16.4% | 16.3% | 8.5% | **9%** | 251 | [5%-12%] |
| Overall Patients | | 241 | 515 | 709 | 800 | 791 | 283 | 59 |  | **2863** |  |

Note: Patient counts are not mutually exclusive.

**Table S2: Hospitalization in All the Patients Having SCD Diagnosis and Inpatient Visit (12-Month Follow-up period)**

|  | **Overall** | | **VOCs** | |  |
| --- | --- | --- | --- | --- | --- |
|  | **Time to First Hospitalization (in Days)** | **Annual Rate of Hospitalization** | **Time to First Hospitalization (in Days)** | **Annual Rate of Hospitalization** |  |
|  |  |  |  |  |  |
|  |  |  |  |  |  |
| **N (patient count)** | 80 | | 37 | |  |
| **Mean** | 129.9 | 1.7 | 158.3 | 1.6 |  |
| **Median** | 109.5 | 1.0 | 162.0 | 1.0 |  |
| **SD** | 107.8 | 1.4 | 121.3 | 1.1 |  |
| **Minimum** | 1.0 | 1.0 | 2.0 | 1.0 |  |
| **Maximum** | 361.0 | 8.0 | 363.0 | 5.0 |  |
| **Q1 (lower quartile)** | 39.0 | 1.0 | 48.0 | 1.0 |  |
| **Q3(upper quartile)** | 198.0 | 2.0 | 253.0 | 2.0 |  |
|  |  |  |  |  |  |

Note:

Q1 (lower quartile) - Q1 is the median (the middle) of the lower half of the data.

Q3 (upper quartile) - Q3 is the median (the middle) of the upper half of the data.

Abbreviations: N: Number of patients; SD: Standard deviation; VOC: Vaso-occlusive crisis

**Table S3: Healthcare Resource Utilization and Costs by Visit Type (6-Month Baseline Period)**

| **Baseline All-Cause Healthcare Resource Utilization and Costs** | | | | |
| --- | --- | --- | --- | --- |
|  | **SCD Patients** | | **SCD Patients (All Age Groups) With VOC Episodes in Index Period** | |
| Number of patients in 6-month baseline | 2863 | | 1211 | |
| Number of claims in 6-month baseline | 12722 | | 4812 | |
| ***Healthcare Utilization: Number of Visits (Claims)*** | | | | |
| Overall |  | |  | |
| N (patient counts) | 2863 | | 1211 | |
| Total | 12722 | | 4812 | |
| Mean | 4.4 | | 4.0 | |
| SD | 3.9 | | 3.5 | |
| Median | 3.0 | | 3.0 | |
| Minimum | 1.0 | | 1.0 | |
| Maximum | 37.0 | | 25.0 | |
| Q1 (lower quartile) | 2.0 | | 2.0 | |
| Q3(upper quartile) | 6.0 | | 5.0 | |
| ***Inpatient Visits*** | | | | |
| N (patient counts) | 488 | | 204 | |
| Total | 680 | | 274 | |
| Mean | 1.4 | | 1.3 | |
| SD | 0.9 | | 0.8 | |
| Median | 1.0 | | 1.0 | |
| Minimum | 1.0 | | 1.0 | |
| Maximum | 6.0 | | 6.0 | |
| Q1 (lower quartile) | 1.0 | | 1.0 | |
| Q3(upper quartile) | 1.0 | | 1.0 | |
| ***Outpatient Visits*** | | | | |
| N (patient count) | 2833 | | 1193 | |
| Total | 12042 | | 4538 | |
| Mean | 4.3 | | 3.8 | |
| SD | 3.7 | | 3.4 | |
| Median | 3.0 | | 3.0 | |
| Minimum | 1.0 | | 1.0 | |
| Maximum | 34.0 | | 25.0 | |
| Q1 (lower quartile) | 2.0 | | 2.0 | |
| Q3(upper quartile) | 6.0 | | 5.0 | |
| ***Healthcare Costs (Reported in US$)*** | | | | |
|  |  | **Overall costs** |  |  |
| N (patient count) | 2863 | | 1211 | |
| Total |  | 271369.5 |  | 109046.8 |
| Mean |  | 94.8 |  | 90.1 |
| SD |  | 139.5 |  | 152.6 |
| Median |  | 55.4 |  | 48.8 |
| Minimum |  | 0.0 |  | 0.0 |
| Maximum |  | 2536.2 |  | 2536.2 |
| Q1 (lower quartile) |  | 27.7 |  | 25.0 |
| Q3(upper quartile) |  | 107.9 |  | 100.9 |
| ***Inpatient Cost*** | | | | |
| N (patient counts) | 488 | | 204 | |
| Total |  | 60302.3 |  | 27868.8 |
| Mean |  | 123.6 |  | 136.6 |
| SD |  | 198.1 |  | 240.1 |
| Median |  | 65.3 |  | 70.4 |
| Minimum |  | 0.0 |  | 0.0 |
| Maximum |  | 2402.6 |  | 2402.6 |
| Q1 (lower quartile) |  | 33.1 |  | 36.8 |
| Q3(upper quartile) |  | 127.9 |  | 141.6 |
| ***Outpatient Cost*** | | | | |
| N (patient count) | 2833 | | 1193 | |
| Total |  | 211067.3 |  | 81178.0 |
| Mean |  | 74.5 |  | 68.1 |
| SD |  | 82.7 |  | 83.5 |
| Median |  | 49.9 |  | 43.4 |
| Minimum |  | 0.0 |  | 0.0 |
| Maximum |  | 933.1 |  | 933.1 |
| Q1 (lower quartile) |  | 25.5 |  | 23.2 |
| Q3(upper quartile) |  | 91.4 |  | 81.2 |

Note:

Currency conversion rates

Source for conversion of Cedi to US$ currency: https://www.unitconverters.net/currency/ghs-to-usd.htm; Accessed on 11August2022 14:52:0

1 Ghanaian Cedi=0.1132855133United States dollar (currency values in US$ rounded off to one decimal point)

Q1 (lower quartile) - Q1 is the median (the middle) of the lower half of the data.

Q3 (upper quartile) - Q3 is the median (the middle) of the upper half of the data

Abbreviations: CEDI: Ghana cedi; N: Number of patients; SCD: Sickle cell disease; SD: Standard deviation; US$: United States Dollar; VOC: Vaso-occlusive crisis

**Table S4: Healthcare Resource Utilization and Costs by Activity Type (12-Month Follow-up)**

| Sickle Cell Disease | | | | |
| --- | --- | --- | --- | --- |
|  | **SCD-Related Healthcare Resource Utilization and Cost** | | **VOC-Related Healthcare Resource Utilization and Cost** | |
| Number of patients in the 12-month follow- up | 406 | | 206 | |
| Number of claims in the 12-month follow-up | 971 | | 359 | |
| Healthcare Utilization: Number of Visits (Claims) | | | | |
| *Drugs* | | | | |
| N (patient count) | 271 | | 187 | |
| Total | 504 | | 317 | |
| Mean | 1.9 | | 1.7 | |
| SD | 1.8 | | 1.5 | |
| Median | 1.0 | | 1.0 | |
| Minimum | 1.0 | | 1.0 | |
| Maximum | 16.0 | | 11.0 | |
| Q1 (lower quartile) | 1.0 | | 1.0 | |
| Q3(upper quartile) | 2.0 | | 2.0 | |
| *Consumables* | | | | |
| N (patient count) | 76 | | 65 | |
| Total | 120 | | 105 | |
| Mean | 1.6 | | 1.6 | |
| SD | 1.2 | | 1.2 | |
| Median | 1.0 | | 1.0 | |
| Minimum | 1.0 | | 1.0 | |
| Maximum | 8.0 | | 8.0 | |
| Q1 (lower quartile) | 1.0 | | 1.0 | |
| Q3(upper quartile) | 2.0 | | 2.0 | |
| *Procedures* | | | | |
| N (patient count) | 356 | | 181 | |
| Total | 651 | | 275 | |
| Mean | 1.8 | | 1.5 | |
| SD | 1.9 | | 1.2 | |
| Median | 1.0 | | 1.0 | |
| Minimum | 1.0 | | 1.0 | |
| Maximum | 15.0 | | 8.0 | |
| Q1 (lower quartile) | 1.0 | | 1.0 | |
| Q3 (upper quartile) | 2.0 | | 2.0 | |
| *Services* | | | | |
| N (patient count) | 346 | | 199 | |
| Total | 758 | | 339 | |
| Mean | 2.2 | | 1.7 | |
| SD | 2.3 | | 1.5 | |
| Median | 1.0 | | 1.0 | |
| Minimum | 1.0 | | 1.0 | |
| Maximum | 21.0 | | 11.0 | |
| Q1 (lower quartile) | 1.0 | | 1.0 | |
| Q3(upper quartile) | 2.0 | | 2.0 | |
| *Others* | | | | |
| N (patient count) | 64 | | 49 | |
| Total | 96 | | 78 | |
| Mean | 1.5 | | 1.6 | |
| SD | 1.1 | | 1.2 | |
| Median | 1.0 | | 1.0 | |
| Minimum | 1.0 | | 1.0 | |
| Maximum | 6.0 | | 6.0 | |
| Q1 (lower quartile) | 1.0 | | 1.0 | |
| Q3(upper quartile) | 1.0 | | 2.0 | |
| Healthcare Costs (Reported in US$) | | | | |
| *Drugs* | | | | |
| N (patient count) | 271.0 | | 187.0 | |
| Total |  | 8108.3 |  | 6850.8 |
| Mean |  | 29.9 |  | 36.6 |
| SD |  | 87.6 |  | 102.0 |
| Median |  | 8.7 |  | 10.9 |
| Minimum |  | 0.0 |  | 0.0 |
| Maximum |  | 1048.1 |  | 1048.1 |
| Q1 (lower quartile) |  | 3.6 |  | 5.0 |
| Q3(upper quartile) |  | 24.4 |  | 32.6 |
| *Consumables* | | | | |
| N (patient count) | 76.0 | | 65.0 | |
| Total |  | 1333.8 |  | 1169.3 |
| Mean |  | 17.5 |  | 18.0 |
| SD |  | 43.5 |  | 46.1 |
| Median |  | 6.4 |  | 6.7 |
| Minimum |  | 0.4 |  | 0.4 |
| Maximum |  | 352.9 |  | 352.9 |
| Q1 (lower quartile) |  | 3.4 |  | 3.4 |
| Q3(upper quartile) |  | 13.6 |  | 13.6 |
| *Procedures* | | | | |
| N (patient count) | 356.0 | | 181.0 | |
| Total |  | 8612.5 |  | 3472.6 |
| Mean |  | 24.2 |  | 19.2 |
| SD |  | 36.6 |  | 24.2 |
| Median |  | 12.1 |  | 11.3 |
| Minimum |  | 0.0 |  | 0.0 |
| Maximum |  | 434.4 |  | 206.2 |
| Q1 (lower quartile) |  | 7.1 |  | 7.4 |
| Q3(upper quartile) |  | 24.3 |  | 21.5 |
| *Services* | | | | |
| N (patient count) | 346.0 | | 199.0 | |
| Total |  | 11188.9 |  | 7215.6 |
| Mean |  | 32.3 |  | 36.3 |
| SD |  | 85.2 |  | 100.4 |
| Median |  | 11.3 |  | 9.9 |
| Minimum |  | 0.0 |  | 3.4 |
| Maximum |  | 984.5 |  | 867.2 |
| Q1 (lower quartile) |  | 6.2 |  | 5.7 |
| Q3(upper quartile) |  | 27.2 |  | 22.1 |
| *Others* | | | | |
| N (patient count) | 64.0 | | 49.0 | |
| Total |  | 671.5 |  | 514.5 |
| Mean |  | 10.5 |  | 10.5 |
| SD |  | 13.5 |  | 12.5 |
| Median |  | 5.1 |  | 6.8 |
| Minimum |  | 0.0 |  | 0.3 |
| Maximum |  | 65.5 |  | 65.5 |
| Q1 (lower quartile) |  | 2.4 |  | 2.8 |
| Q3(upper quartile) |  | 13.8 |  | 13.8 |

Note:

Currency conversion rates

Source for conversion of Cedi to US$ currency: https://www.unitconverters.net/currency/ghs-to-usd.htm; Accessed on 03August2022 18:30:0

1 Cedi=0.1156860978 United States dollar (currency values in US$ rounded off to one decimal point)

Q1 (lower quartile) - Q1 is the median (the middle) of the lower half of the data

Q3 (upper quartile) - Q3 is the median (the middle) of the upper half of the data

Consumables - Medical/surgical supplies; Procedures - Medical/surgical procedures or diagnostic investigations; Services - Consultation or hospital services; Others - Administrative services

Abbreviations: CEDI: Ghana cedi; N: Number of patients; SCD: Sickle cell disease; SD: Standard deviation; US$: United States Dollar; VOC: Vaso-occlusive crisis

**Table S5: Specialities Visited by Patients with SCD (January 2015-March 2021)**

|  | | | | |
| --- | --- | --- | --- | --- |
|  | **Number of Patients** | **% of Patients** |  |  |
| SCD-diagnosed patient count | 2863 |  |  |  |
| SCD-diagnosed patient with specialty visit | 2408 | 84% |  |  |
| SCD-diagnosed patient claims | 4116 |  |  |  |
| **Specialty** | **Number of Patients** | **% of Patients** | **Number of claims** | **% of Claims** |
| GP consultation | 1810 | 75.2% | 2867 | 69.7% |
| Pediatrics | 357 | 14.8% | 618 | 15.0% |
| Gynecology consultation | 154 | 6.4% | 227 | 5.5% |
| Specialist consultation | 137 | 5.7% | 252 | 6.1% |
| Review (GP consultation) | 94 | 3.9% | 116 | 2.8% |
| Specialist consultation (physician) | 52 | 2.2% | 76 | 1.8% |
| Review (specialist consultation) | 30 | 1.2% | 32 | 0.8% |
| Specialist consultation(surgeon) | 23 | 1.0% | 27 | 0.7% |
| Specialist consultation (dietician) | 8 | 0.3% | 8 | 0.2% |
| Specialist consultation (orthopedics) | 8 | 0.3% | 8 | 0.2% |
| Specialist consultation (urologist) | 4 | 0.2% | 4 | 0.1% |
| Specialist consultation(cardiologist) | 3 | 0.1% | 3 | 0.1% |
| General OPD – child | 2 | 0.1% | 2 | 0.0% |
| Specialist consultation (dermatologist) | 1 | 0.0% | 1 | 0.0% |

Note: Numbers are not mutually exclusive.

Abbreviations: GP: General Physician; OPD: Outpatient department; SCD: Sickle cell disease

GP consultation: Patient consulted general practitioner for evaluation and treatment

Review (GP consultation): Patient consulted general practitioner for review or follow-up of previously reported condition

Specialist consultation: Patient consulted a specialist for evaluation or treatment Here the type of specialist (pediatrician/gynecologist/urologist/cardiologist/dermatologist/physician/orthopedician) consulted by patient was not specified.

Review (Specialist consultation): Patient consulted specialist for review or follow-up of treatment of the previously condition reported. Here the type of specialist whom the patient consulted during follow-up visit was not specified.

General OPD-child: Pediatric patient consulted general practitioner for evaluation and treatment.

Pediatrics: This refers to consultation with a pediatrician

**Table S6: Diagnostic Investigations Conducted on Patients With SCD (January 2015-March 2021)**

| **Diagnostic Investigation** | | | | |
| --- | --- | --- | --- | --- |
|  | **Number of Patients** | **% of Patients** |  |  |
| SCD-diagnosed patient count | 2863 |  |  |  |
| SCD patients who underwent diagnostic tests | 2759 | 96% |  |  |
| SCD patient claims | 4185 |  |  |  |
| **Diagnostic Investigation** | **Number of Patients** | **% of Patients** | **Number of claims** | **% of Claims** |
| FBC (Automation) | 2114 | 76.6% | 3013 | 72.0% |
| Hb electrophoresis | 1644 | 59.6% | 1775 | 42.4% |
| BF for malarial parasites | 1427 | 51.7% | 1975 | 47.2% |
| Sickling test | 1142 | 41.4% | 1229 | 29.4% |
| Routine urine examination | 1097 | 39.8% | 1455 | 34.8% |
| Blood grouping | 823 | 29.8% | 864 | 20.6% |
| G6PD | 607 | 22.0% | 637 | 15.2% |
| Fasting blood sugar/random blood sugar | 529 | 19.2% | 588 | 14.1% |
| Widal test | 465 | 16.9% | 556 | 13.3% |
| HIV screening/retro | 409 | 14.8% | 432 | 10.3% |
| Routine stool examination | 397 | 14.4% | 419 | 10.0% |
| HBSAg/ HBV | 324 | 11.7% | 340 | 8.1% |
| VDRL | 323 | 11.7% | 342 | 8.2% |
| BUN and Creatinine | 219 | 7.9% | 265 | 6.3% |
| Malaria card test (Antigen) (Dipstick assay/RDT) | 211 | 7.6% | 269 | 6.4% |
| LFT | 200 | 7.2% | 245 | 5.9% |
| ESR | 162 | 5.9% | 175 | 4.2% |
| Urine C/S | 145 | 5.3% | 150 | 3.6% |
| Toxoplasmosis | 140 | 5.1% | 141 | 3.4% |
| Rubella | 138 | 5.0% | 139 | 3.3% |
| Pelvic scan | 134 | 4.9% | 150 | 3.6% |
| X-ray one region | 120 | 4.3% | 122 | 2.9% |
| FBC (Auto) and film comment | 109 | 4.0% | 146 | 3.5% |
| Lipid profile | 105 | 3.8% | 107 | 2.6% |
| Hb estimation | 91 | 3.3% | 130 | 3.1% |
| Serum uric acid | 79 | 2.9% | 82 | 2.0% |
| RFT/KFT | 74 | 2.7% | 91 | 2.2% |
| *H Pylori* (*Helicobacter Pylori* test) | 65 | 2.4% | 65 | 1.6% |
| Pregnancy test | 55 | 2.0% | 55 | 1.3% |
| Blood for C/S | 54 | 2.0% | 61 | 1.5% |
| Typhi dot | 53 | 1.9% | 60 | 1.4% |
| HbA1c | 51 | 1.8% | 53 | 1.3% |
| ECG | 48 | 1.7% | 48 | 1.1% |
| Coombs test (direct) | 44 | 1.6% | 44 | 1.1% |
| Abdominopelvic scan | 41 | 1.5% | 43 | 1.0% |
| Hepatitis C screening/HCV | 37 | 1.3% | 40 | 1.0% |
| Reticulocyte count | 36 | 1.3% | 72 | 1.7% |
| Blood Grouping and antibody screen | 35 | 1.3% | 35 | 0.8% |
| Rheumatoid factor | 34 | 1.2% | 35 | 0.8% |
| Reticulocyte count (retics) | 30 | 1.1% | 49 | 1.2% |

Abbreviations: BUN: Blood urea nitrogen; C/S: Culture and sensitivity aerobic; ECG: Electrocardiogram; ESR: Erythrocyte sedimentation rate; FBC: Full blood count; G6PD: Glucose-6-phosphate dehydrogenase; Hb: Hemoglobin; HbA1c: Glycosylated hemoglobin; HBSA: Hepatitis B surface antigen; HBV: Hepatitis B virus; HCV: Hepatitis C virus; HIV: Human immunodeficiency virus; KFT: Kidney function test; LFT: Liver function test; RDT: Rapid diagnostic test; RFT: Renal function test; SCD: Sickle cell disease; VDRL: Venereal Disease Research Laboratory
